# IgG4-related disease-like lesion arising within a well-differentiated liposarcoma/an atypical lipomatous tumor: two cases report

**DOI:** 10.3389/fonc.2025.1596391

**Published:** 2025-08-29

**Authors:** Naoki Oike, Tomoaki Torigoe, Akira Ogose, Takashi Ariizumi, Tomohiro Miyazaki, Yudai Murayama, Taku Homma, Hajime Umezu, Takuya Watanabe, Hiroyuki Kawashima

**Affiliations:** ^1^ Division of Orthopedic Surgery, Graduate School of Medical and Dental Sciences, Niigata University, Niigata, Japan; ^2^ Department of Orthopaedic Oncology and Surgery, Saitama Medical University International Medical Center, Saitama, Japan; ^3^ Department of Orthopedics, Uonuma Kikan Hospital, Niigata, Japan; ^4^ Division of Musculoskeletal Oncology and Orthopedic Surgery, Tochigi Cancer Center, Tochigi, Japan; ^5^ Department of Diagnostic Pathology, Saitama Medical University International Medical Center, Saitama, Japan; ^6^ Department of Pathology, Graduate School of Medical and Dental Sciences, Niigata University, Niigata, Japan

**Keywords:** inflammatory, IgG4, IgG-4 related disease, soft tissue sarcoma, well differentiated liposarcoma, murine double minute 2, case report

## Abstract

**Introduction:**

Well-differentiated liposarcoma (WDLPS) is a locally aggressive soft tissue sarcoma characterized by the amplification of *MDM2* and/or *CDK4*. IgG4-related disease (IgG4-RD) is a rare fibroinflammatory disease characterized by elevated serum IgG4 levels and histologically fibrous tissues with IgG4-positive immune cell infiltration. Although IgG4-RD often mimics other diseases, including malignant neoplasms, the association between IgG4-RD and WDLPS has not been fully elucidated. Here, we present two cases of IgG4-RD occurring within WDLPS.

**Case presentation:**

A 56-year-old man presented with a left retroperitoneal mass, and a 64-year-old woman presented with a non-tender mass in her right thigh. Magnetic resonance imaging revealed that the tumors were composed of adipose and non-adipose areas with homogeneous enhancement. A biopsy revealed that the tumor consisted of adipose cells of various sizes and fibrous tissue with IgG4-positive cell infiltration. *MDM2* amplification was detected using fluorescence *in situ* hybridization in both the adipose and fibrous areas. In addition, serum IgG4 levels were elevated in both patients, and IgG4-RD within the WDLPS were diagnosed. Wide resection was performed as radical treatment. After resection, the serum IgG4 level decreased to normal. No recurrence or metastasis has been observed for >19 months.

**Conclusion:**

Our cases highlight the importance of careful biopsy for the accurate diagnosis of patients with IgG4-RD within WDLPS. Moreover, although the main treatment for IgG4-RD is systemic corticosteroids, IgG4-RD within WDLPS should be treated with surgical resection.

## 1 Introduction

Atypical lipomatous tumor/well-differentiated liposarcoma (ALT/WDLPS) is a locally aggressive mesenchymal neoplasm characterized by amplification of MDM2 and/or CDK4 (1). According to the 2020 World Health Organization (WHO) classification of soft tissue and bone tumors, WDLPS is the most common subtype of adipocytic malignancies, accounting for approximately 40–45% of liposarcomas (1).

IgG4-related disease (IgG4-RD) was first described by Hamano et al. in patients with sclerosing cholangitis and elevated serum IgG4 levels (2). IgG4-RD is a chronic immune-mediated inflammatory condition typically characterized by varying degrees of fibrosis, elevated serum IgG4 levels, and tissue infiltration by IgG4-positive plasma cells (2).

IgG4-RD often mimics other diseases, including malignant neoplasm (3, 4). Thus, it is important to differentiate IgG4-RD from other malignancies because the treatments for these diseases are often quite different.

However, to the best of our knowledge, IgG4-RD has not been previously described in the context of ALT/WDLPS. Here, we present two interesting cases of IgG4-RD like lesion occurring within WDLPS.

## 2 Case presentation

### 2.1 Case 1

**Figure 1:**
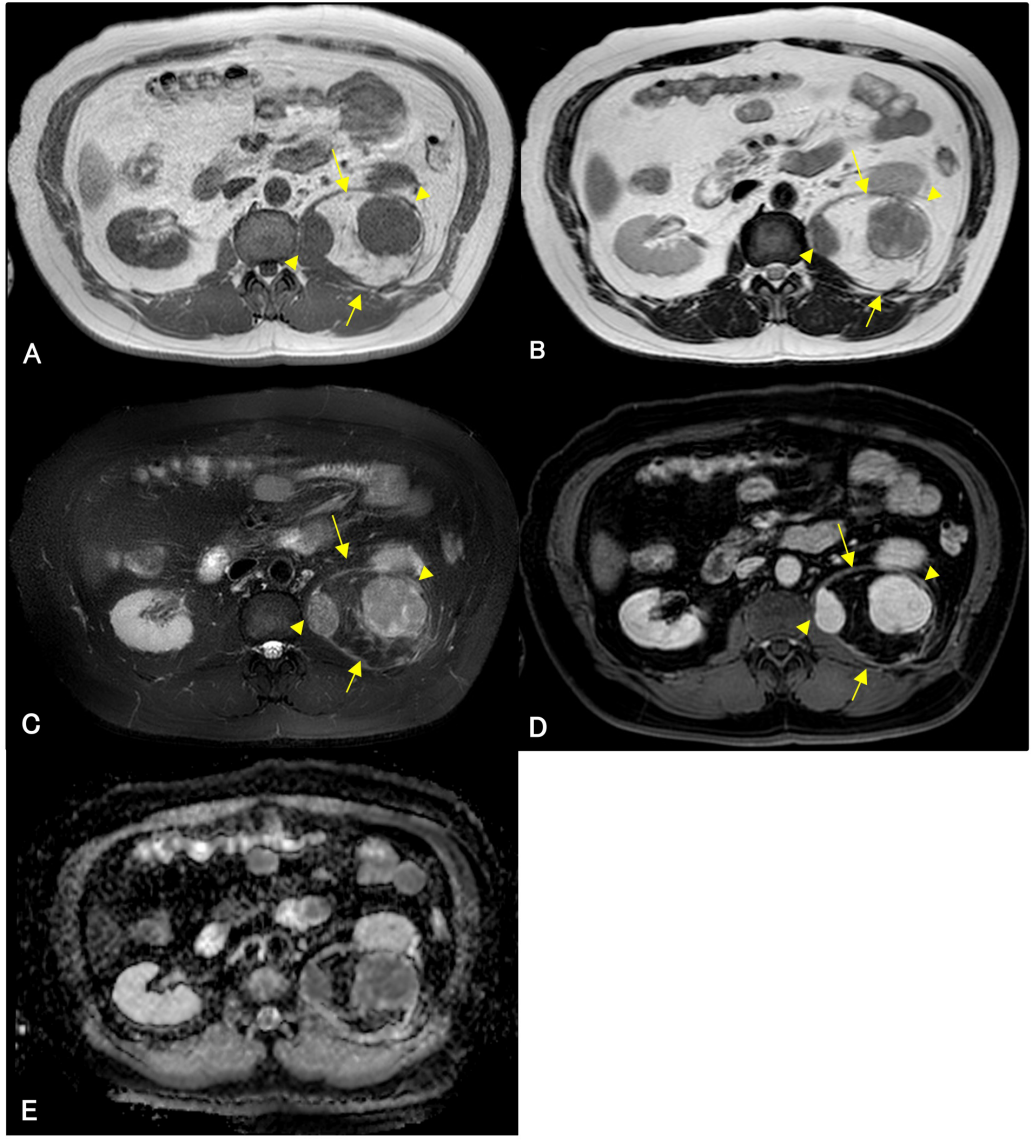
Magnetic resonance imaging (MRI) of an adipose mass in the left retroperitoneum of a 56-year-old man. Isointense lesions (arrowheads) are observed within a high-signal mass (arrows) on well-defined T1-weighted images **(A)**. These lesions demonstrate mildly high intensity (arrowheads) on T2-weighted and T2-weighted fat-suppression imaging **(B, C)**. The isointense areas show gadolinium enhancement **(D)**, and low signal intensity on Apparent Diffusion Coefficient (ADC) map **(E)**. High-intensity lesions (arrows) on T1-weighted imaging **(A)** and T2-weighted imaging **(B)** are suppressed on T2-weighted fat-suppression imaging **(C)**. The lesion shows no gadolinium enhancement **(D)**.

**Figure 2:**
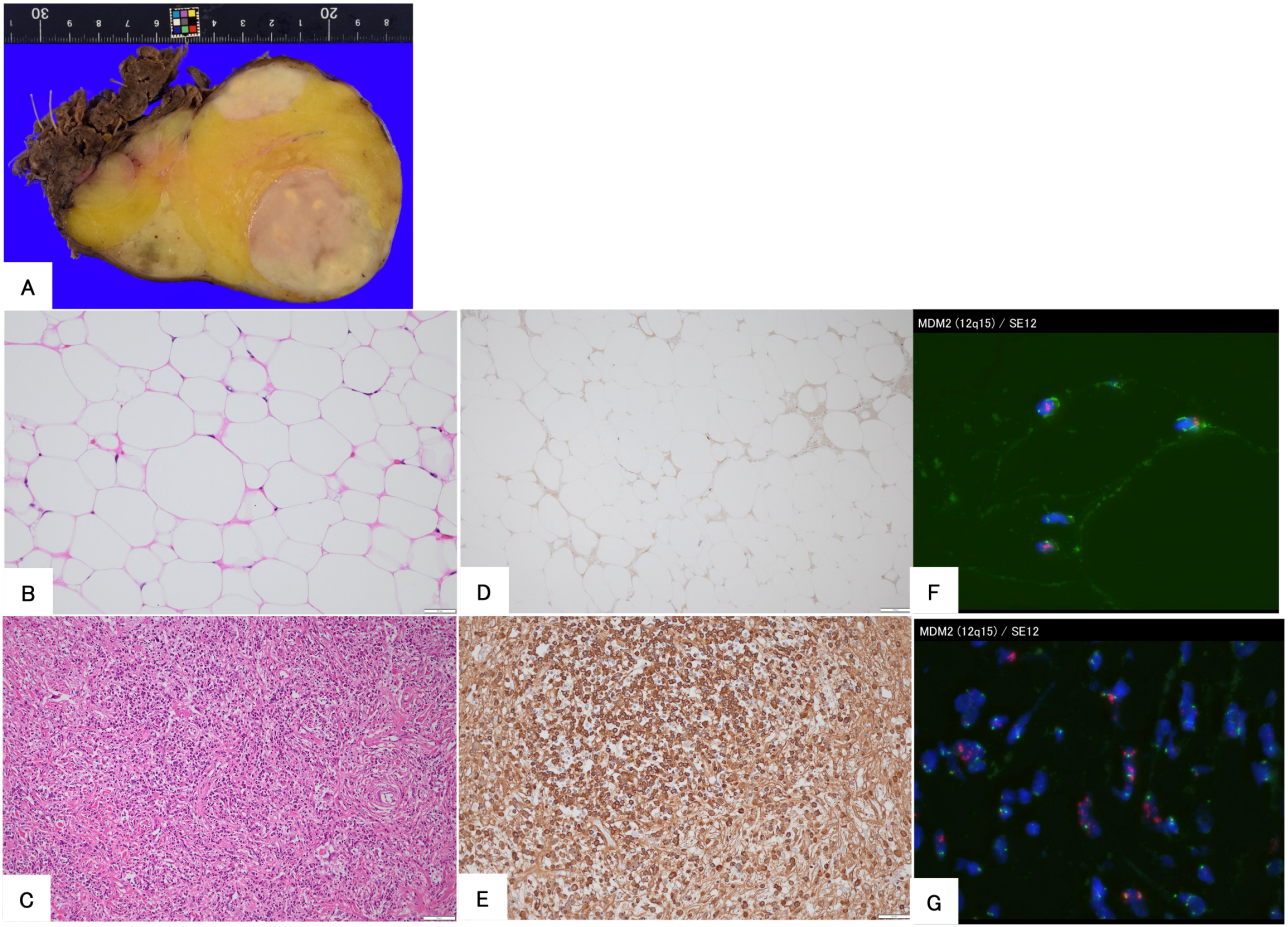
Histopathological findings of the surgical specimen in a 56-year-old man. The gross appearance of the resected specimen demonstrates that the tumor consists of yellow and white areas with well-defined borders **(A)**. Hematoxylin and eosin staining demonstrated that the tumor is composed of mature adipose tissue **(B)** and fibrous areas with immune cell infiltration **(C)**. Immunohistochemical staining for MDM2 shows positive nuclear staining **(D)**, and IgG4 is positive in plasma cells in the fibrous area **(E)**. Fluorescence *in situ* hybridization demonstrates the amplification of MDM2 in both adipose **(F)** and fibrous areas **(G)**.

A 56-year-old man underwent computed tomography (CT) for the preoperative examination of hemorrhoids. CT revealed a left retroperitoneal mass, and there were no lesions in other organs except for the retroperitoneal mass. The patient was referred to our hospital for further evaluation. The patient was an asymptomatic human immunodeficiency virus carrier. The patient demonstrated no fever up. On physical examination, palpation revealed no left abdominal masses and no lymphadenopathy. CT showed a well-defined 4.3 cm × 4.3 cm × 4.2 cm intramuscular mass with a fat-density area and high-density area with homogeneous contrast enhancement in the left psoas muscle. Magnetic resonance imaging (MRI) demonstrated that the tumor consisted of a well-defined non-adipose area with isointensity on T1-weighted imaging and mild hyperintensity on T2-weighted imaging within an adipose area of high intensity on both T1-weighted and T2-weighted imaging. High-intensity areas on both T1- and T2-weighted images were suppressed on T2-weighted fat-suppression imaging. Homogeneous gadolinium enhancement was observed in the non-adipose areas. Non-adipose areas demonstrated low signal intensity on Apparent Diffusion Coefficient (ADC) map (**Figure 1**). Based on imaging findings, dedifferentiated liposarcoma (DDLPS) was suspected, and biopsy specimens were obtained separately from both the non-adipose and adipose areas using a CT-guided biopsy. Increased IgG4-positive cells were identified by immunohistochemistry in the non-adipose area, and mature adipose tissue of varying sizes was observed in the adipose area. Immunohistochemical expression of MDM2 and CDK4 was observed in the nuclei of adipose tissue. Fluorescence *in situ* hybridization (FISH) demonstrated amplification of the *MDM2* gene amplification. Laboratory tests revealed elevated serum IgG4 (277 mg/dL, normal values 11- 121mg/dL) and C-reactive protein (0.426 mg/dL, normal values <0.14mg/dL) levels. Based on these findings, IgG4-RD within WDLPS was suspected, and wide resection was performed. Macroscopically, the tumor was predominantly yellow lipomatous with two well-defined white nodules, consistent with the imaging findings (**Figure 2A**). Histologically, the tumor was composed of an adipose component of varying sizes with septal structures and atypical nuclei (**Figure 2B**), whereas the other components were predominantly collagen fibers and fibrous atypical cells with chronic inflammatory cell infiltration (**Figure 2C**). Immunohistochemistry demonstrated positive expression of MDM2 (**Figure 2D**) and CDK4 in the adipose component. Numerous IgG4-positive cells were found in the fibrous component of the infiltrating inflammatory cells, but not in the lipomatous component (**Figure 2E**). FISH analysis demonstrated *MDM2* amplification in both adipose and fibrous areas (**Figure 2F, G**).

After the surgery, the serum IgG4 level decreased to normal (99 mg/dL), and the patient showed no evidence of local recurrence, distant metastasis, or re-elevation of serum IgG4 levels 19 months postoperatively.

### 2.2 Case 2

**Figure 3:**
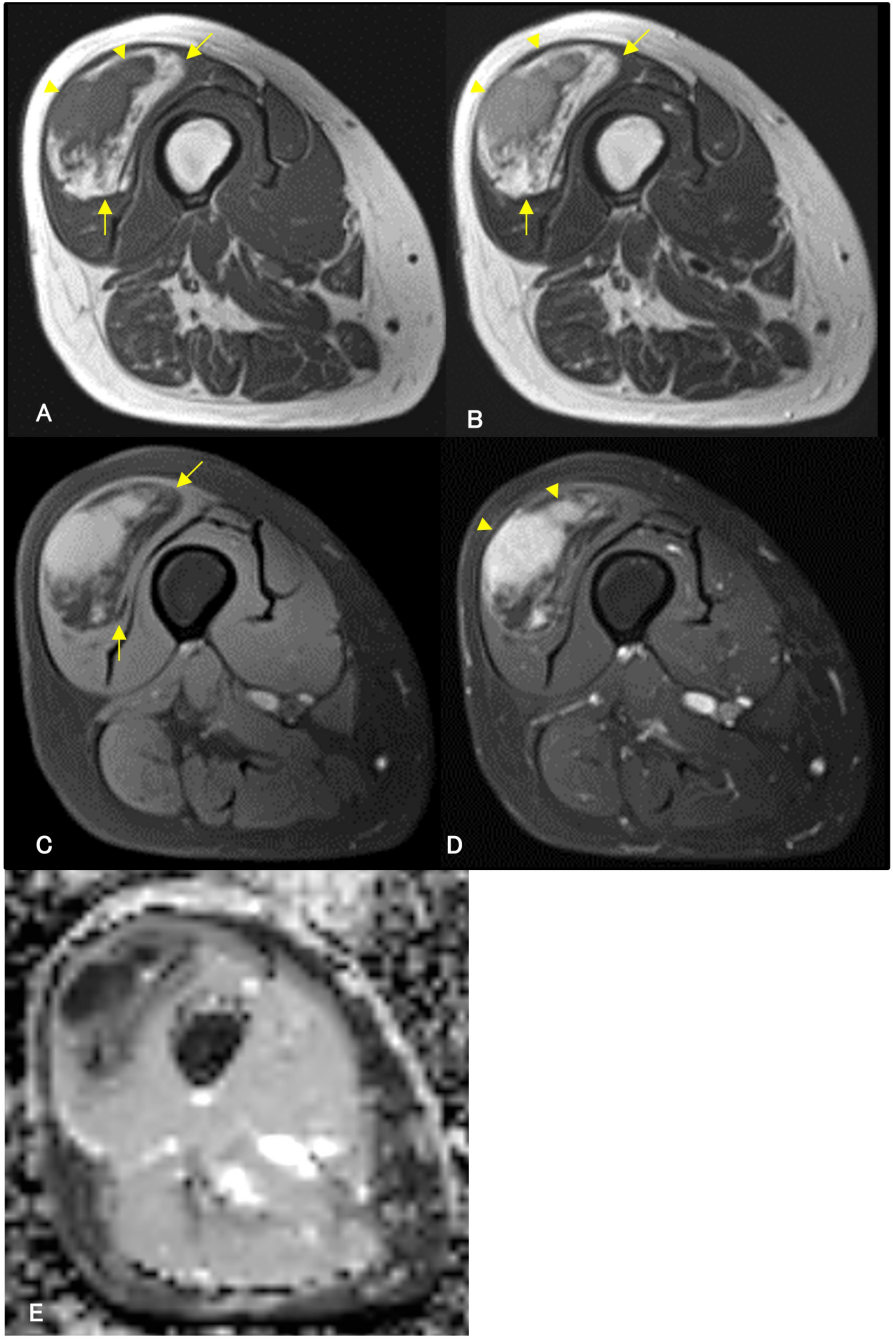
Contrast-enhanced magnetic resonance imaging (MRI) of a 64-year-old woman. A contrast-enhanced MRI reveals a well-defined mass in the right vastus lateralis muscle. MRI demonstrates an isointense area (arrowheads) on T1-weighted imaging **(A)** and mild hyperintensity (arrowheads) on T2-weighted imaging **(B)** within the high-intensity area (arrows) on both T1- and T2-weighted imaging **(A, B)**. High-intensity areas on both T1-weighted and T2-weighted images are suppressed (arrows) in T2-weighted fat-suppressed imaging **(C)**, suggesting a non-adipose component within the adipose tumor. The non-adipose component demonstrates homogeneous enhancement with gadolinium arrowheads, **(D)**, and low signal intensity on Apparent Diffusion Coefficient (ADC) map **(E)**.

**Figure 4:**
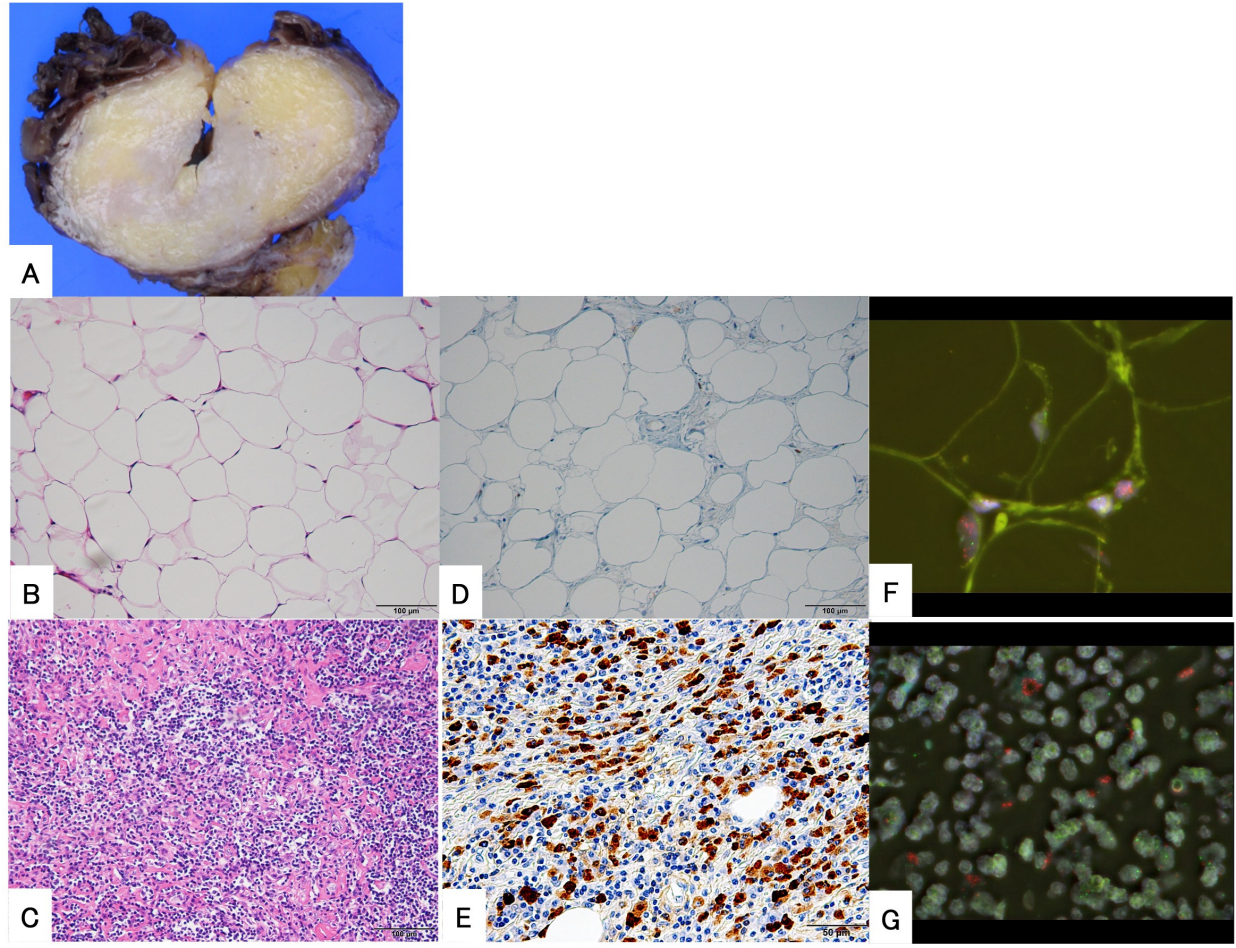
Histopathological findings of the surgical specimen from a 64-year-old woman. The gross appearance of the tumor revealed that it was composed of yellow, fat-like components and white, fibrous components with ill-defined borders **(A)**. The tumor is composed of mature adipose tissue **(B)** and fibrous areas with immune cell infiltration **(C)** with indistinct borders. Immunohistochemical examination shows positive expression of MDM2 in the nucleus **(D)** and demonstrates numerous IgG4-positive plasma cells in the fibrous area **(E)**. Fluorescence *in situ* hybridization demonstrates the amplification of MDM2 in both adipose **(F)** and fibrous areas **(G)**.

A 64-year-old woman noticed a painless mass in her right thigh 7 years prior to presentation. She was referred to our hospital because the mass had been growing rapidly for a year. The patient demonstrated no fever up. Physical examination revealed a non-tender mobile mass in her right thigh, but no lymphadenopathy. MRI demonstrated a well-defined 2.5 cm × 5.4 cm × 9.5 cm mass in her right vastus lateralis muscle (**Figure 3**). MRI showed that the tumor was composed of a non-adipose area with isointensity on T1-weighted imaging and mild hyperintensity on T2-weighted imaging within an adipose mass of high intensity on both T1- and T2-weighted imaging. Unlike in Case 1, the border was ill-defined, but the non-adipose area demonstrated homogeneous gadolinium enhancement, as in Case 1. ADC map showed lower signal intensity in non-adipose areas (**Figure 3**). During core needle biopsy, a non-adipose component was targeted to obtain specimens because we initially suspected a DDLPS. On biopsy, fibrous tissue with inflammatory cell infiltration and adipose tissue without atypia were observed, and the diagnosis from the needle biopsy was IgG4-RD. The serum IgG4 level was elevated to 138 mg/dL; however, the C-reactive protein level was within the normal range (0.02 mg/dL. After consultation with a rheumatologist, the patient was diagnosed with atypical IgG4-RD. This discrepancy led us to perform an open biopsy for a definitive diagnosis. In the open biopsy specimens, variably sized adipose cells and fibrous tissue with IgG4-positive inflammatory cell infiltration were observed. Based on these histological findings, ALT with IgG4-RD was diagnosed. Subsequently, wide resection was successfully performed as curative treatment. The tumor’s gross appearance was a mixture of yellow fat-like and white fibrous components (**Figure 4A**). Histological examination revealed that the tumor consisted of adipose tissue with irregularly sized adipocytes (**Figure 4B**) and fibrous tissue with dense lymphocyte infiltration. Atypical stromal cells were present in the fibrous tissue. The inflammatory cells consisted mainly of plasma cells and lymphocytes (**Figure 4C**).

Immunohistochemical examination revealed the nuclear expression of MDM2 in both adipose cells (**Figure 4D**) and spindle cells. IgG4-positive plasma cells were also found in the fibrous tissue area, and the IgG4-IgG ratio was approximately 50%. No IgG4-positive cells were observed in the adipose are. These results met the histological criteria for IgG4-RD (**Figure 4E**). FISH revealed *MDM2* amplification in both fibrous and lipomatous areas (**Figures 4F, G**).

After the surgery, the serum IgG4 level decreased to normal (42 mg/dL). The patient had no evidence of local recurrence, distant metastasis, or re-elevation of serum IgG4 levels 29 months postoperatively.

## 3 Discussion

IgG4-RD is a recently recognized chronic fibroinflammatory disorder related to immunomodulation. The underlying pathophysiology of IgG4-RD has not been fully elucidated. The working group of the Japanese IgG4 team established comprehensive diagnostic criteria for IgG4-RD in 2011 (5) and revised the criteria for IgG4-RD in 2020 (6). These criteria consist of three items; if the patient meets all three criteria, the diagnosis is considered definite for IgG4-RD (6). Both our patients fulfilled the following criteria: (a) Nodules within the adipose tumor were detected by MRI, (b) serum IgG4 level was > 135 mg/dL, and (c) histological examination revealed that lymphocytes and plasma cells infiltrated the fibrous tissue, and the ratio of IgG4-positive cells was > 40%. Therefore, our patients met all three items of the revised comprehensive criteria and were diagnosed with definite IgG4-RD.

On the other hand, ALT/WDLPS is the most common subtype of liposarcoma and can be divided morphologically into three main subtypes: lipoma-like, sclerosing, and inflammatory subtypes (1). Inflammatory ALT/WDLPS is the rarest subtype and is mostly found in the retroperitoneum. Amplification of *MDM2* and/or *CDK4* is considered a hallmark genetic feature of ALT/WDLPS, and the detection of *MDM2* and/or *CDK4* amplification using quantitative real-time polymerase chain reaction, FISH, or immunohistochemical expression of MDM2 and/or CDK4 is used for the definitive diagnosis of WDLPS (1). In our cases, both patients demonstrated overexpression of the MDM2 protein, which was assessed using immunohistochemistry, and amplification of the *MDM2* gene, which was assessed using FISH. These findings led to the diagnosis of ALT/WDLPS. Our patients met all three criteria for IgG4-RD, and MDM2 amplification was found in both adipose and fibrous areas, suggesting that IgG4-RD occurred within ALT/WDLPS.

It could be that infiltrating immune cells in the inflammatory subtype of ALT/WDLPS demonstrate IgG4 positivity. However, Reagh et al. reported a patient with WDLPS who was initially diagnosed with an inflammatory pseudotumor (7). Another study reported a patient with DDLPS that was initially considered an inflammatory myofibroblastic tumor (8). Notably, in both cases, there was no increase in IgG4-positive cells in inflammatory fibrous tissue. These reports suggest that intratumoral immune cells do not always produce IgG4 in liposarcomas.

In contrast, Harne et al. reported a patient with a retroperitoneal mass that was initially diagnosed with IgG4-RD, but the final diagnosis was WDLPS (9). Unfortunately, because the study did not describe the details of the imaging or histological findings, it was difficult to determine whether their patient was similar to ours. However, taken together with these reports, we considered that the fibrous tumor with immune cell infiltration within the ALT/WDLPS in our two patients was IgG4-RD.

IgG4-RD can affect multiple organs and tissues throughout the body, including the retroperitoneum, thigh and other soft tissue (6, 10). Therefore, it is important to differentiate IgG4-RD from other diseases because the treatments for other diseases, such as malignant neoplasms, are quite different. As mentioned above, some cases have been reported in which a patient was initially diagnosed with an IgG4-RD but was eventually diagnosed with a neoplasm (9), and the reverse has also been observed (4, 11). In our cases, it is important to differentiate between DDLPS and ALT/WDLPS because DDLPS is a more aggressive malignant tumor that requires more extensive resection. In general, MRI is an important imaging modality for diagnosing soft tissue tumors. Recently, the apparent diffusion coefficient (ADC) map has garnered attention as a quantitative imaging tool for characterizing soft tissue tumors, and soft tissue sarcomas tend to exhibit lower ADC values reflecting higher cellularity (12). On the other hand, a low signal on the ADC map is often seen in patients with IgG4-RD, suggesting restricted diffusion due to inflammation and fibrosis (13, 14). ^18^F-fluorodeoxyglucose Positron Emission Tomography/Computed Tomography (PET/CT) is another useful modality for diagnosis, organ involvement, guided biopsy, and treatment response assessment of IgG4-RD (15). FDG accumulates in IgG4-RD lesions because these lesions involve significant infiltration of lymphocytes and IgG4-positive plasma cells. This leads to enhanced glucose metabolism in the affected tissues. Unfortunately, however, malignant soft tissue neoplasm also demonstrated high FDG uptake (16). Thus, there is overlap in imaging findings between DDLPS and IgG4-RD within ALT/WDLPS. Since it is extremely difficult to differentiate IgG4-RD from other malignant tumor based on imaging alone, histopathological examination plays a crucial role in diagnosis (11). However, it is important to obtain an adequate sample for accurate diagnosis. Indeed, in the patient in Case 1, the diagnosis of IgG4-RD within WDLPS was made from the initial biopsy because the samples were obtained from both adipose and non-adipose areas. On the other hand, since the pathological diagnosis from the needle biopsy for the patient in Case 2 was only IgG4-RD, the initial biopsy targeted only the non-adipose area. From the second biopsy, we obtained a diagnosis of IgG4-RD within ALT because we obtained samples from both adipose and non-adipose areas. The reason for this is that we initially considered to be able to differentiate ALT from DDLPS only from non-adipose area. Although, compared to DDLPS, it seems to be quite rare that IgG4-RD arise in ALT/WDLPS, the clinical features of IgG4-RD, such as symptoms and imaging examinations, are non-specific (10). Thus, measuring serum IgG4 levels during the initial evaluation may also help suspect IgG4-RD within ALT/WDLPS.

Emerging evidence suggests that IgG4-RD increases the incidence of malignant tumors, particularly pancreatic cancer and lymphoma (17, 18). Regarding sarcomas, Joo et al. reported a patient with gastrointestinal clear cell sarcoma associated with IgG4-RD (19). As we discussed, the authors mentioned the possibility of the coexistence of primary gastrointestinal clear cell sarcoma and IgG4-related fibrotic disease, as well as an IgG4-related abnormal immune response specific to gastrointestinal clear cell sarcoma. Additionally, some case reports demonstrated infiltration of IgG4-positive lymphocytes in patients with sarcoma (9, 20). We have several hypotheses regarding the etiology of our cases. First, IgG4-related abnormal immune response occurred in ALT/WDLPS like Joo’s case. Second, immune cells in the inflammatory variants of ALT/WDLPS sometimes produce IgG4, and these two cases may represent rare variants of inflammatory ALT/WDLPS. Lastly, additional mutations could occur in ALT/WDLPS, similar to DDLPS. Determining the true pathology is difficult because both IgG4-RD and inflammatory ALT/WDLPS are rare, and pathogenic mutation of IgG4-RD remains unclear (21). Further analysis is necessary to understand the pathology of this disease. For example, the proportion of inflammatory variants of ALT/WDLPS that produce IgG4, or gene analysis from both adipose area and non-adipose area should probably be examined,

The treatment of IgG4-RD mainly involves systemic corticosteroids and, in some cases, rituximab (22). In contrast, the treatment of WDLPS involves surgical resection with wide margins. In Harne’s case, although the serum IgG4 level decreased with systemic prednisolone treatment, the tumor grew. Following a second biopsy, the diagnosis was changed from IgG4-RD to WDLPS, and the patient underwent wide resection (9). In our cases, both patients underwent curative wide resection as definitive treatment, and the serum IgG4 levels decreased to normal after the surgeries without additional treatment. Neither patient developed local recurrence or re-elevation of serum IgG4 levels. Patients with IgG4-RD after resection may benefit from adjuvant systemic treatment (11). However, some patients with IgG4-RD, including a soft tissue case, have not experienced recurrence after resection without systemic treatment (10, 11, 23). Although the number of cases is small and further study is needed, surgical resection may sometimes be a curative treatment for patients with IgG4-RD. Determining disease activity is possible through monitoring of the serum IgG4 level (22). However, to the best of our knowledge, there are no reports on methods for monitoring recurrence in patients after complete resection. Considering that IgG4 does not increase in all patients with IgG4-RD, and that ALT/WDLPS demonstrates high local recurrence rate especially in the retroperitoneum (1). We consider that the patients with IgG4-RD within ALT/WDLPS should probably monitor both serum IgG4 and imaging modalities such as CT or MRI.

## 4 Conclusions

In conclusion, we presented two patients with IgG4-RD within WDLPS. When lipomatous tumors which are suspected to be ALT/WLPS or DDLPS on radiological examination, IgG4-RD should probably be considered in the differential diagnosis. Assessment of serum IgG4 levels at the time of initial evaluation may help distinguish between these diseases. In addition, our cases highlight the importance of careful biopsy for a definitive diagnosis. To avoid repeated biopsies, biopsy specimens should be obtained from both adipose and non-adipose areas. Wide resection may be a curative treatment for patients with IgG4-RD within WDLPS.

## Data Availability

The raw data supporting the conclusions of this article will be made available by the authors, without undue reservation.

## References

[1] Board Whocte. Soft tissue and bone tumours. 5th ed. Geneva, Switzerland: World Health Organization International Agency for Research on Cancer (2020). p. 607.

[2] Hamano H Kawa S Horiuchi A Unno H Furuya N Akamatsu T. High serum IgG4 concentrations in patients with sclerosing pancreatitis. N Engl J Med. (2001) 344:732–8. doi: 10.1056/NEJM200103083441005, PMID: 11236777

[3] Pacyna RR Cipriani NA Mathew MS Kim JS. IgG4-related disease mimicking gynecologic Malignancy. Gynecol Oncol Rep. (2023) 45:101137. doi: 10.1016/j.gore.2023.101137, PMID: 36714372 PMC9879761

[4] Bertoglio P Viti A Paiano S Assante LR Bogina GS Pomari C. IgG4-related disease: a new challenging diagnosis mimicking lung cancer. Interact Cardiovasc Thorac Surg. (2019) 28:410–2. doi: 10.1093/icvts/ivy279, PMID: 30295799

[5] Umehara H Okazaki K Masaki Y Kawano M Yamamoto M Saeki T. Comprehensive diagnostic criteria for IgG4-related disease (IgG4-RD), 2011. Mod Rheumatol. (2012) 22:21–30. doi: 10.3109/s10165-011-0571-z 22218969

[6] Umehara H Okazaki K Kawa S Takahashi H Goto H Matsui S. The 2020 revised comprehensive diagnostic (RCD) criteria for IgG4-RD. Modern Rheumatol. (2021) 31:529–33. doi: 10.1080/14397595.2020.1859710, PMID: 33274670

[7] Reagh JJ Eckstein RP Selinger CI Evans J Gill AJ. Liposarcoma masquerading as an inflammatory pseudotumor: a case report. J Med Case Rep. (2016) 10:64. doi: 10.1186/s13256-016-0858-y, PMID: 26987706 PMC4797231

[8] Cai M Siew CCH Tay TKY Tan GHC. Dedifferentiated liposarcoma with a rare presentation of disseminated intraperitoneal sarcomatosis: A case report. Int J Surg Case Rep. (2019) 60:331–5. doi: 10.1016/j.ijscr.2019.06.051, PMID: 31280066 PMC6612661

[9] Harne PS Soni U Albustamy A Rivera AS Zamir A. Liposarcoma masquerading as immunoglobulin G4-related disease. ACG Case Rep J. (2024) 11:e01249. doi: 10.14309/crj.0000000000001249, PMID: 38179263 PMC10766308

[10] Creze M Boussebaa S Lazure T Briand S Court C. IgG4-related disease: rare presentation as a soft-tissue mass in the thigh of an adolescent. Skeletal Radiol. (2020) 49:155–60. doi: 10.1007/s00256-019-03250-9, PMID: 31165193

[11] Kawasaki T Ichikawa J Onohara K Kanno S Wako M Taniguchi N. IgG4-related disease with subcutaneous involvement and the associated diagnostic challenges with MRI. Skeletal Radiol. (2025) 54:1147–51. doi: 10.1007/s00256-024-04768-3, PMID: 39085476 PMC11953168

[12] Choi YJ Lee IS Song YS Kim JI Choi KU Song JW. Diagnostic performance of diffusion-weighted (DWI) and dynamic contrast-enhanced (DCE) MRI for the differentiation of benign from Malignant soft-tissue tumors. J Magn Reson Imaging. (2019) 50:798–809. doi: 10.1002/jmri.26607, PMID: 30663160

[13] Kim B Kim JH Byun JH Kim HJ Lee SS Kim SY. IgG4-related kidney disease: MRI findings with emphasis on the usefulness of diffusion-weighted imaging. Eur J Radiol. (2014) 83:1057–62. doi: 10.1016/j.ejrad.2014.03.033, PMID: 24768583

[14] Tang CSW Sivarasan N Griffin N. Abdominal manifestations of IgG4-related disease: a pictorial review. Insights Imaging. (2018) 9:437–48. doi: 10.1007/s13244-018-0618-1, PMID: 29696607 PMC6108972

[15] Naik M Hesni S Tamimi A Hameed M Tomlinson J Poo S. Imaging manifestations of IgG4-related disease. Clin Radiol. (2023) 78:555–64. doi: 10.1016/j.crad.2023.03.003, PMID: 37217396

[16] Katal S Gholamrezanezhad A Kessler M Olyaei M Jadvar H. PET in the diagnostic management of soft tissue sarcomas of musculoskeletal origin. PET Clin. (2018) 13:609–21. doi: 10.1016/j.cpet.2018.05.011, PMID: 30219191 PMC7466822

[17] Yu T Wu Y Liu J Zhuang Y Jin X Wang L. The risk of Malignancy in patients with IgG4-related disease: a systematic review and meta-analysis. Arthritis Res Ther. (2022) 24:14. doi: 10.1186/s13075-021-02652-2, PMID: 34986892 PMC8728936

[18] Tang H Yang H Zhang P Wu D Zhang S Zhao J. Malignancy and IgG4-related disease: the incidence, related factors and prognosis from a prospective cohort study in China. Sci Rep. (2020) 10:4910. doi: 10.1038/s41598-020-61585-z, PMID: 32188869 PMC7080711

[19] Joo M Chang SH Kim H Gardner JM Ro JY. Primary gastrointestinal clear cell sarcoma: report of 2 cases, one case associated with IgG4-related sclerosing disease, and review of literature. Ann Diagn Pathol. (2009) 13:30–5. doi: 10.1016/j.anndiagpath.2008.10.003, PMID: 19118779

[20] Wei S Han Y Hou Y Hu J. Primary pulmonary angiosarcoma was misdiagnosed as IgG4-related disease by needle biopsy: A challenging case report. Indian J Pathol Microbiol. (2025) 68:415–7. doi: 10.4103/ijpm.ijpm_794_23, PMID: 38847188

[21] Ishikawa Y Terao C. Genetic analysis of IgG4-related disease. Mod Rheumatol. (2020) 30:17–23. doi: 10.1080/14397595.2019.1621000, PMID: 31104539

[22] Lanzillotta M Mancuso G Della-Torre E. Advances in the diagnosis and management of IgG4 related disease. Bmj. (2020) 369:m1067. doi: 10.1136/bmj.m1067, PMID: 32546500

[23] Ishii K Hisa T Kudo A Osera S Shinohara T Tomori A. A resected case of focal autoimmune pancreatitis with pancreatic duct wall thickening representing periductal lymphoplasmacytic infiltrate. Clin J Gastroenterol. (2021) 14:1278–85. doi: 10.1007/s12328-021-01428-0, PMID: 34091821

